# *In Vivo* Near-Infrared Imaging of Fibrin Deposition in Thromboembolic Stroke in Mice

**DOI:** 10.1371/journal.pone.0030262

**Published:** 2012-01-17

**Authors:** Yi Zhang, Shufeng Fan, Yuyu Yao, Jie Ding, Yu Wang, Zhen Zhao, Lei Liao, Peicheng Li, Fengchao Zang, Gao-Jun Teng

**Affiliations:** Jiangsu Key Laboratory of Molecular and Functional Imaging, Department of Radiology, Zhongda Hospital, Medical School of Southeast University, Nanjing, China; Ohio State University, United States of America

## Abstract

**Objectives:**

Thrombus and secondary thrombosis plays a key role in stroke. Recent molecular imaging provides *in vivo* imaging of activated factor XIII (FXIIIa), an important mediator of thrombosis or fibrinolytic resistance. The present study was to investigate the fibrin deposition in a thromboembolic stroke mice model by FXIIIa–targeted near-infrared fluorescence (NIRF) imaging.

**Materials and Methods:**

The experimental protocol was approved by our institutional animal use committee. Seventy-six C57B/6J mice were subjected to thromboembolic middle cerebral artery occlusion or sham operation. Mice were either intravenously injected with the FXIIIa-targeted probe or control probe. *In vivo* and *ex vivo* NIRF imaging were performed thereafter. Probe distribution was assessed with fluorescence microscopy by spectral imaging and quantification system. MR scans were performed to measure lesion volumes *in vivo*, which were correlated with histology after animal euthanasia.

**Results:**

*In vivo* significant higher fluorescence intensity over the ischemia-affected hemisphere, compared to the contralateral side, was detected in mice that received FXIIIa-targeted probe, but not in the controlled mice. Significantly NIRF signals showed time-dependent processes from 8 to 96 hours after injection of FXIIIa-targeted probes. *Ex vivo* NIRF image showed an intense fluorescence within the ischemic territory only in mice injected with FXIIIa-targeted probe. The fluorescence microscopy demonstrated distribution of FXIIIa-targeted probe in the ischemic region and nearby micro-vessels, and FXIIIa-targeted probe signals showed good overlap with immune-fluorescent fibrin staining images. There was a significant correlation between total targeted signal from *in vivo* or *ex vivo* NIRF images and lesion volume.

**Conclusion:**

Non-invasive detection of fibrin deposition in ischemic mouse brain using NIRF imaging is feasible and this technique may provide an in vivo experimental tool in studying the role of fibrin in stroke.

## Introduction

Stroke is the second leading cause of death worldwide and ranks first for disablement. It is estimated that one sixth of all human beings will suffer at least one stroke in their life time [1], [2], [3]. Thromboembolic events are responsible for approximately 80% of human stroke [4], [5]. Dynamic imaging of thrombus and secondary thrombosis in vivo can play a key role in understanding pathophysiology of stroke after the occlusion of an intracranial artery and mechanism of thrombolytic therapy.

With recent advances in near infrared (NIR)-activatable fluorescent probe technology, near infrared fluorescence (NIRF) imaging has been used in vivo to observe protease activity in a murine stroke model [6], [7]. NIRF imaging essentially depends on the probes emitting in the NIR spectrum band width between 650–900 nm in which biological tissues display low absorption and imaging shows high target-to-background ratios due to reduced autofluorescence [8]. This technology offers several advantages including high sensitivity and being radiation free, and it can be performed with comparatively simple and inexpensive instrumentation [7]. Although the major disavantage of weak penetration with NIRF imaging hinders its clinical application currently, it is a useful technique for investigating the pathological process of diseases. Moreover, it may be potential clincial use by intraoperation [9] or interventional procedures in the future.

Experimentally, activated factor XIII (FXIIIa), a thrombin activated tetrameric transglutaminase, is an important mediator of thrombosis or fibrinolytic resistance [10], [11]. Recently, a near-infrared fluorescent probe(A15) has been developed that is recognized by FXIIIa [12]. It covalently binds to fibrin, providing unique means to image the activity of the FXIIIa as well as to visualize thrombus in vivo [11], [13].

In this study, we investigated the potential use of the FXIIIa-targeted probe for in vivo fibrin deposition in ischemic brain tissue in a mouse thromboembolic stroke model. This study aims to answer specifically: (a) whether the fluorescent probe targeting fibrin could be used to identify and quantify fibrin deposition in regions of cerebral ischemia in vivo and ex vivo, (b) whether the dynamic process of fibrin deposition can be observed in vivo using this fluorescent probe, (c) whether NIRF signal intensity for fibrin deposition in regions of cerebral ischemia using this fluorescent probe correlates with ischemic lesion volume.

## Results

### *In vivo* and *ex vivo* NIRF imaging

**Figure 1:**
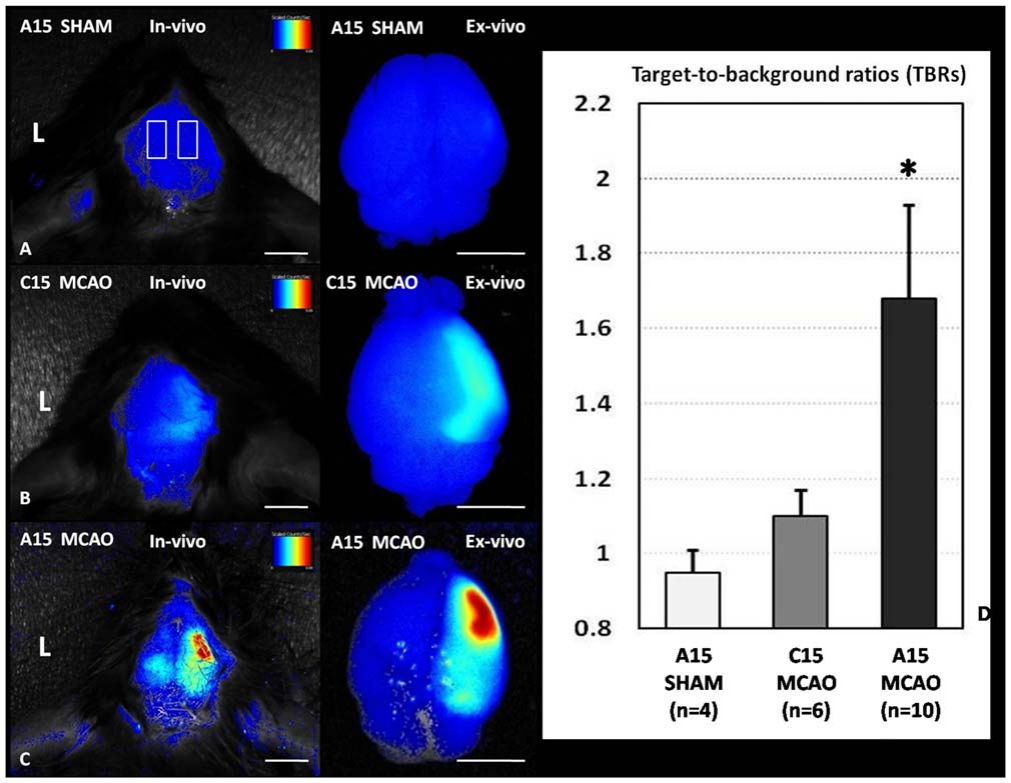
Representative NIRF imaging of different groups 24 h after infusion of the fluorescent probe. In vivo NIRF imaging (A, Left) or ex vivo NIRF imaging of the mouse brains removed from the skull (A, Right) showed no difference between the hemispheres in sham-operated mice injected with the A15 probe. The rectangular boxes represented the ROIs placed over the right and left hemisphere. There was only slight increase of fluorescence intensities in the injected hemisphere compared with the contralateral side in MCAO mice injected with the C15 control probe (in vivo NIRF imaging, B, Left; and ex vivo NIRF imaging, B, Right). Strong fluorescence was seen over the ipsilateral side of MCAO mice injected with the A15 probe shown by in vivo NIRF imaging (C, Left) or ex vivo NIRF imaging (C, Right). The images were normalized on the color scaling bar. Target-to-background ratios (TBRs) were calculated from ROI analyses of noninvasive NIRF images in different groups as shown in Fig D. Only the MCAO mice receiving the A15 probe showed significantly higher TBRs (*P<0.05 versus SHAM). Bar in (A–C) = 5 mm.

In vivo NIRF imaging (Figure 1A, Left) or ex vivo NIRF imaging of the mouse brains removed from the skull (Figure 1A, Right) showed no difference between right and left hemispheres in sham-operated mice injected with the FXIIIa-targeted NIRF imaging probe (A15). The rectangular regions give an example of the Regions of interest (ROIs) placed over the right and left hemisphere. Slightly higher fluorescence intensities over the injected hemisphere compared with the contralateral side were detected in thromboembolic middle cerebral artery occlusion (MCAO) mice injected with the control C15 probe (only different than A15 at position 14 amino-acid residue) by both the in vivo NIRF image (Figure 1B, Left) and the ex vivo NIRF image (Figure 1B, Right). Strong fluorescence was seen over the ipsilateral side of MCAO mice injected with the A15 probe on the in vivo NIRF image (Figure 1C, Left) and the ex vivo NIRF image (Figure 1C, Right). The corresponding ex vivo NIRF images showed equally low fluorescence intensities over both hemispheres in brains of sham-operated mice that were injected with A15.

Sham-operated mice that received A15 and MCAO mice that received C15 both showed statistically significant lower Target-to-background ratios (TBRs) compared to MCAO mice that received A15 (0.95±0.06 and 1.10±0.07 versus 1.68±0.25, respectively; P<0.05) (Figure 1 D).

### Time courses of *in vivo* NIRF imaging of MCAO mice after injection of A15

**Figure 2:**
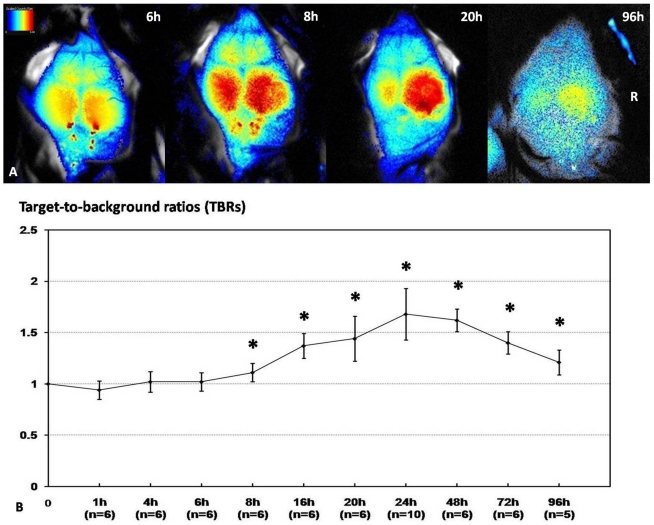
Time courses of in vivo NIRF imaging of MCAO mice injected with A15 at 6 h, 8 h, 20 h and 96 h (A). Quantification of target-to-background ratios (TBRs) at different time points (From 0–96 h post injection of A15) was shown in B. The group that were significantly different were compared with 0 h (TBRs = 1). (*P<0.05 versus time 0).

The results from noninvasive NIRF images obtained at different time points after injection of A15 are shown in Figure 2. In mice imaged with NIRF at 1 hour, 4 hours and 6 hours after injection of A15, there was no significant difference observed between the fluorescence intensities measured over the ipsilateral, ischemic hemisphere compared with the contralateral side (TBRs: 0.94±0.09, 1.02±0.10 and 1.02±0.09, respectively). In contrast, significantly higher fluorescence signals were detected over the ipsilateral hemisphere compared with the contralateral side in mice imaged with NIRF at 8 hours, 16 hours, 20 hours, 24 hours, 48 hours, 72 hours and 96 hours (Figure 2A) after injection of A15 (TBRs: 1.11±0.09, 1.37±0.12, 1.44±0.22, 1.68±0.25, 1.62±0.11, 1.40±0.11 and 1.21±0.12, respectively; n = 6, P<0.05). Significantly TBRs changes were clearly detected initially at 8 hours after injection of A15, and it showed time-dependent increase until at 24 hours after injection. TBRs values also decrease gradually at 48, 72 and 96 hours (Figure 2B).

### NIRF imaging of brain slices and histological examination

**Figure 3:**
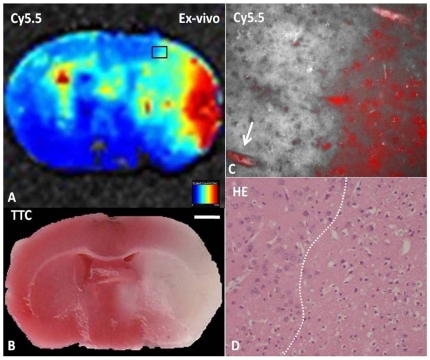
Distribution of the injected A15 probe 24 h after cerebral ischemic induction. Areas with high fluorescence intensities were observed over the ischemic region in MCAO mice that received A15 (A), corresponding to the pallor in TTC staining (B). High magnification of the boxed region in A was shown in C (C, 40×). Intense and diffuse Cy5.5 fluorescence from A15 was observed in the ischemic area of the cortex and nearby micro-vessels (↑), whereas fluorescence was scarcely visible in the nonischemic areas. The border between the ischemic and nonischemic area was clearly delineated after TTC (B) and hematoxylin and eosin staining (D).

A lesion was delineated by triphenyltetrazolium chloride (TTC) staining at 24 hours after injection of clots in MCAO mice injection of NIRF probes (Figure 3). In sham-operated mice receiving A15, weak fluorescence was evenly distributed over the brain slice. Areas of low fluorescence signals, corresponding to the pallor in TTC staining, were seen in MCAO mice injected with C15. Areas with high fluorescence signals were observed over the ischemic region in MCAO mice that received A15. Ex vivo NIRF imaging of brain slices, bilateral hemispheres showed differential fluorescence intensities in the brains of MCAO mice 24 h after injection of the A15 probe. Higher fluorescence signals were observed in the ischemic lesion, corresponding to the pallor in TTC staining, compared to areas surrounding the lesion (Figure 3 A, B). The highest fluorescence signals were detected in the cortex of the ischemic hemisphere mainly (Figure 3A). The volumes were calculated from A15 signal larger than from TTC ischemia lesion (A15: 76.48±7.14% and TTC: 64.76±10.75%). Fluorescence microscopy was used to assess the distribution of the A15 probe with higher magnification. Intense, diffuse Cy5.5 fluorescence from A15 was observed in the ischemic area of the cortex and nearby micro-vessels (Figure 3C), whereas fluorescence is scarcely visible in the non-ischemic area. The lesion border is delineated after hematoxylin and eosin staining (Figure 3D).

### Fluorescence Microscopy and Immunohistochemistry

**Figure 4:**
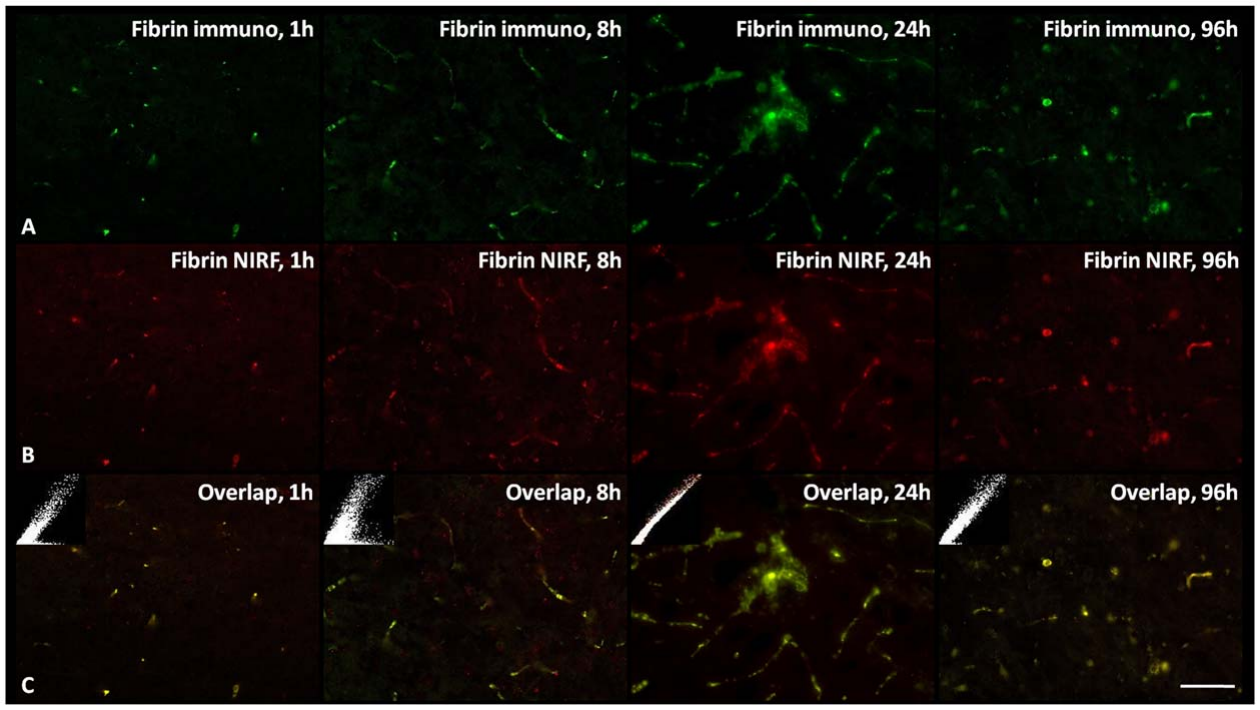
Fluorescence images of brain sections showed the ipsilateral cortex of MCAO mice at 1, 8, 24 and 96 hours after intravenous injection of A15. The green signals showed the staining with FITC-labeled antibodies against fibrin (A), and the red signals (NIRF channel) displayed the distribution of the injected Cy5.5-labeled probes (B). The embedded scatter gram in the upper left corner of each image (C) showed good overlap of colocalization analysis (C). (40×, Bar = 50 µm).

In MCAO mice injected with A15 probe at 1, 8, 24 and 96 hours, Cy5.5 fluorescence was detected in the ischemic core. The fluorescence from injected probes at different time points (Figure 4B) revealed a similar pattern (Figure 4C) to the antibody staining against the fibrin (Figure 4A). The embedded scatter gram in the upper left corner of each image showed good overlap of colocalization analysis (Figure 4C).

### Relationship between targeted signals from NIRF imaging and ischemic lesion volume

**Figure 5:**
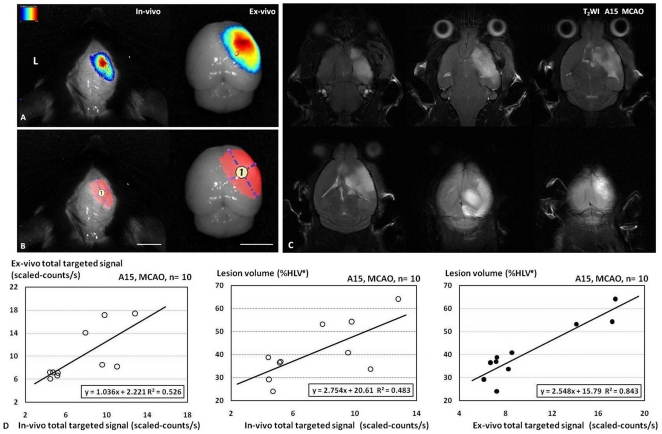
Quantitative analysis of targeted signals from in vivo and ex vivo NIRF imaging of the mouse brains removed from the skull and lesion volume of T_2_WI (%HLV^e^). The targeted signals of in vivo and ex vivo NIRF images are separated from background signals by multispectral imaging technology (A). ROI was selected by threshold segmentation (B). Fig. 5C shows T_2_WI of MCAO mice 24 h after injection of A15, and area of hyperintensity delineates ischemia lesion. A significant correlation is observed between the total targeted signal values of in vivo NIRF images (R^2^ = 0.526, P<0.05) and the total targeted signal values of ex vivo NIRF images of MCAO mice injected with the A15 probe (D, Left). A significant correlation is observed between the value of total targeted signal from in vivo NIRF imaging (R^2^ = 0.483, P<0.05) or from ex vivo NIRF imaging (R^2^ = 0.843, P<0.05) and lesion volume of T2WI (%HLV^e^) (D, Middle, Right). Bar in (A–D) = 5 mm.

The targeted signals were separated and calculated by multispectral imaging technology from MCAO mice that received A15 (n = 10) (Figure 5 A, B). Linear regression analysis showed a significant correlation (R^2^ = 0.526, P<0.05) between the total targeted signal values of non-invasive NIRF images and the total targeted signal values of ex vivo NIRF images of MCAO mice injected with the A15 probe (Figure 5D, Left).

No abnormal signal was seen in T_2_-weighted image (T_2_WI) of sham-operated mice. At 24 h post injection of thrombus, hyper intensity was detected in ipsilateral hemisphere of MCAO mice (Figure 5C). In MCAO mice injected with the A15 probe (n = 10), linear regression analysis demonstrated a significant correlation (R^2^ = 0.483, P<0.05) between the value of total targeted signal of in vivo NIRF imaging and percent of edema–corrected lesion volume (%HLV^e^) (Figure 5D, Middle). The values of total targeted signal of ex vivo NIRF imaging against %HLV^e^ also showed a linear correlation (R^2^ = 0.843, P<0.05) (Figure 5D, Right).

## Discussion

For the first time, our study demonstrated that a factor involved in secondary fibrin deposition in cerebral ischemia could be specifically and non-invasively revealed using an infrared probe A15, and in vivo NIRF imaging in an animal model.

Microvascular fibrin deposition has been shown to accumulate during early focal cerebral ischemia and reperfusion in the nonhuman primate [14], [15]; and fibrin-containing microthromboemboli were found in acute human ischemic brain [16]. Previous studies have established a link between ischemic brain injuries and reduced antithrombotic mechanism in brain microcirculation in the presence of major stroke risk factors [17], [18]. Thrombosis starts with the activation of thrombin, which converts soluble fibrinogen to fibrin [19].Visualization of fibrin distribution in vivo without sacrificing the experimental animal is highly desirable to study the pathophysiology and treatment of acute stroke. Just like human ischemic stroke. The major advantage of thromboembolic MCAO model used in this experiment is that it is more similar to pathophysiology of ischemic stroke in humans. NIRF imaging using fluorescent probes is a promising technology for visualizing biological events noninvasively in small animals and potentially in clinical settings [20]. An optical probe (A15), which is based on the aminoterminus of alpha-2 antiplasmin and covalently integrated into thrombi by means of the transglutamase activity of FXIIIa, has been specifically developed for NIRF imaging in vivo [10], [11], [13].

Time sequences were distinctively crucial in experimental research of thrombolytic therapy in acute stroke models. Our results confirmed that fibrin deposition after cerebral ischemia was time-dependent, being most prominent at 24 hours [21]. Early fibrin depositions in microvessels at 1 hour (1 h) after cerebral ischemia have, however, been reported [16] and been observed in this study. The A15 probe was tested within these time frames in MCAO mice. At 1 h, 4 h, and 6 h there is no detectable fluorescent activity in affected hemisphere using A15 probe. Strong fluorescence intensities, on the other hand, were clearly observed over the ischemic hemispheres compared with the non-ischemic hemispheres on noninvasive and ex vivo NIRF images at 8 h after injection of A15, and strongest fluorescence intensities were observed at 24 h after injection of A15. The fibrin depositions in microvessels were time-dependent, and it decreased gradually beyond 24 h after injection of A15. Noninvasive NIRF imaging using A15 probe was able to reveal this course in vivo.

The A15 probe was injected in sham-operated mice as controls for activation of the probe unrelated to cerebral ischemia. Low fluorescence intensities were found equally distributed over both ipsilateral and contralateral hemispheres on in vivo NIRF images. To test the specificity of A15, a non-binding of control C15 probe was also injected to MCAO mice. In those mice, there was no difference between hemispheres seen based on in vivo NIRF images using the control C15 probe. When the brains were removed from the skull and imaged with NIRF ex vivo, fluorescence intensities in the affected ispilateral brain tissue were slightly higher than the contralateral brain tissue using the control C15 probe. With C15, the amounts of fluorescent probes in the ischemic tissue were clearly below the in vivo detection limit and were nondetectable with noninvasive NIRF. In the MCAO mice that received the A15 probe, focal high fluorescence was detected in the ischemic region and nearby micro-vessels. In contrast, the signal was rather diffuse and scarcely visible in MCAO mice that received C15. The results suggest the binding of A15 probe is largely attributable to specific affinity of the probe to fibrins in the damaged tissue rather than to damaged tissue with impaired blood– brain barrier. Ex vivo NIRF imaging of brain slices from MCAO mice 24 h after injection of the A15 probe showed that the highest fluorescence signal is concentrated mainly in the cortex of the ischemic hemisphere. Fibrin deposition in the cortex is later than in other regions of ischemic hemisphere. These results support the hypothesis that fibrin deposition in the cortex is later than other places such as the striatum of ischemic hemisphere, because of existence of collateral blood vessels and the penumbra.

Edema-corrected LV calculation on MRI indicated lesion accurately [22]. The values of total targeted signal from in vivo or ex vivo NIRF images and the lesion size calculated using %HLV^e^ of T_2_WI on MRI showed significant correlations. One potential explanation is that micro-vessel obstructions by fibrin deposition in the ischemic region have a close relationship with the irreversibility of the damage from ischemic insults. This is supported by the fact that anticoagulant therapy can diminish lesion volumes of cerebral ischemia [23]. FXIII catalyzes intermolecular cross-linking reactions between fibrin monomers, 2-plasmin inhibitor, and fibronectin. These reactions increase the mechanical strength of the fibrin clot and its resistance to proteolytic degradation, and enhance the assembly of the extracellular matrix [10], [24], [25]. Noncontrolled cross-linking by FXIIIa results in overcross- linked fibrin, and extensive cross-linking of other plasma proteins to fibrin, that could lead to undesired prolonged persistence of thrombi [26]. The stability of fibrin was disclosed indirectly owing to activity of FXIII. The linear correlation between the values of targeted signal in vivo or ex vivo NIRF images and lesion volumes in T_2_WI supports the hypothesis that fibrin depositions in the region of cerebral ischemia play a key role in neuronal damage leading to cerebral infarction.

MRI has played a pivotal role in noninvasive acute stroke research. MRI, however, has difficulty in demonstrating secondary thrombosis in microcirculations directly. More recently, fibrin-specific MR contrast agents have been reported to show coronary and pulmonary clots, as well as cerebral venous sinus thrombosis, and may have the ability to differentiate between the early phases of thrombus formation and organized thrombi in carotid artery. However, the sensitivity using the agent to demonstrate thrombi in microvessels is poor [27], [28]. On the other hand, NIRF imaging has shown excellent sensitivity and specificity. The disadvantage of NIRF imaging technique, on the other hand, is its lack of spatial resolution. In the present study, combining MRI with NIRF imaging allows for localization of biological signal to subtle anatomical structures.

Although fibrinolytic therapies have been used extensively in the treatment of strokes, the role of extravasated fibrin in the rehabilitation of the CNS after the primary neuronal damage caused by cerebral ischemia remains to be elucidated. Fibrin and FXIIIa may regulate cell survival and apoptosis, modulate migration, proliferation and cytokine secretion that play a pivotal role in the orchestration of the immune response [26], [29]. These properties make fibrin a mediator of pathogenic effects that extend beyond blood coagulation. The nervous tissue provides the necessary receptors and intracellular signaling pathways to mediate fibrin-induced cellular responses [30].The activation of an enzyme such as FXIIIa and the deposition of fibrin would be expected to serve as early predictors for the success or failure of a therapeutic regimen in treatment of ischemic injuries to the brain. We have shown that A15 may be used as a non-invasive probe to study fibrin and cellular FXIII function in ischemic stroke. The A15 agent could be expected to have utility in molecular imaging to guide therapy with fibrinolytic or anti-FXIIIa agents, direct or indirect thrombin inhibitors, or other drugs in the molecular passways.

In conclusion, in vivo visualization and quantification of fibrin deposition after cerebral ischemia using NIRF imaging is feasible in the mouse model and may provide an experimental tool in studying the role of fibrin in the pathophysiology of ischemic stroke.

## Materials and Methods

### The synthesis of the FXIIIa-targeted NIRF imaging probe

The chemical synthesis of the FXIIIa-targeted NIRF imaging probe was modified from the previous reference [12]. Briefly, the FXIIIa affinity peptide, Ac-GN13QEQVSPLTLLK24WC, based on the N-terminus of alpha-2-antiplasmin (a2-AP) was synthesized by solid-phase peptide synthesis and then labeled with Cy 5.5 (Molecular Probes, Eugene, OR, USA) via its cysteine side chain. The compound was purified using high-performance liquid chromatography, resulting in the final FXIIIa-targeted NIRF imaging agent (A15). The excitation and emission wavelengths of A15 were 673 and 692 nm, respectively. A control probe modified by replacing glutamine residue at position 14 with an alanine residue to prevent binding to fibrin was also synthesized (C15).

### Animals

This study was approved by Institutional Animal Care and Use Committee (IACUC) of the Medical School of Southeast University (approval ID: SYXK-2010.4987). A total of 76 male C57BL/6J mice (28.5–32 g, Shanghai Laboratory Animal Center, Chinese Academy of Science, China) were used in this study. Animals were housed under standard conditions.

### Animal model

Thromboembolic stroke mouse models were prepared as described previously, with some modifications [23], [31]. Briefly, eight fibrin-rich 48-hr-old homologous cylindrical thrombus of 1.5 mm in length were injected from external carotid artery (ECA) to internal carotid artery (ICA) via a PE0402 (ID = 0.2 mm, OD = 0.38 mm, Anilab, Ningbo, Zhejiang, China) catheter. Sham operation was performed with the same surgical procedures but without injection of the thrombus.

### Experimental design

The experimental mice were divided into two groups randomly (MCAO group, n = 72 and sham group, n = 4). NIRF imaging was either performed at 1, 4, 6, 8, 16, 20, 24, 48, 72 and 96 h (MCAO, n = 6, 6, 6, 6, 6, 6, 10, 6, 6 and 5, respectively) with mice after the injection of A15 probes or after the injection of (A15, sham, n = 4 or C15, MCAO, n = 6) probes at 24 h. The fluorescent compounds were diluted in PBS (5 nmol/100 µL, per mouse) and injected into the tail vein or femoral vein as a bolus immediately after the injection of thrombus. T_2_-weighted MRI images (T_2_WI) were obtained after NIRF imaging at 24 h post thrombus injection. The MCAO mice without ischemic lesion in T_2_WI (n = 1, MCAO 24 h group) or dead mice (n = 2, MCAO 24 h group and 96 h group) were excluded from further analysis. For ex vivo NIRF imaging, the brains were removed under deep anesthesia after MRI, and coronal brain slices of 1-mm thickness were cut in a mice brain matrix using a razor blade.

### *In vivo* NIRF imaging

Mice were fixed in an examining unit, and the skull exposed by a longitudinal skin incision (about 2 cm) in all of the mice. Fluorescent images were obtained with the Maestro in vivo imaging system (CRi, Woburn, MA, USA) at predetermined time points. Two filter sets (Red: excitation, 616–661 nm; Red: emission: 675 nm Long pass; liquid crystal tunable filter (LCTF): 670 nm to 900 nm in 10 nm steps) were used to detect probe-specific fluorescence (from PPCD conjugates) and auto-fluorescence (primarily from the skins and blood vessels). The fluorescent images were then analysed based on their spectral patterns using Maestro 2.10.0 Software (CRi, Woburn, MA, USA).

The fluorescence signals were separated by multispectral imaging technology. The measurement was carried out in grey level integration. ROIs analysis was performed by a person who was blinded to the experimental groups. ROIs were selected by threshold segmentation or manually drawn regions using the same standard. For analysis of time points and group signal changes, ROIs were selected manually by drawing regions on the in vivo NIRF images. Rectangular ROIs were selected over the right and left hemisphere using normalized NIRF in vivo images (Figure 1A, Left). TBRs were defined as: (ROI values from the right hemisphere)/(ROI values from the left hemisphere) [6], [20]. Threshold segmentation of ROI selection for lesion quantification was used for correlative analysis. The total signal (scaled-counts/s) within the ROI was calculated.

### Magnetic Resonance Imaging

Magnetic resonance imaging was performed on a high magnetic field micro-MR research scanner (7.0T Bruker PharmaScans, Bruker Biospin, Ettlingen, Germany). Data acquisition and image processing were performed using a Paravision 5.0 software platform (Bruker Biospin, Ettlingen, Germany). Mice were subjected to isoflurane anesthesia. Respiratory rate and body temperature were monitored using a physiology monitoring unit (Model 1025, SA Instruments Inc, Stony Brook, NY, US), and temperature was maintained within physiologic limits using an animal warming system (MT1025, Bruker Biospin, Ettlingen, Germany). T_2_-weighted images were generated using a two-dimensional turbo spin-echo sequence (TR/TE = 2,862/20 msecs, RARE factor 8, 4 averages). Ten axial slices with a slice thickness of 1 mm, a field of view of 2.0×2.0 cm and a matrix of 256×256 were positioned over the brain excluding the olfactory bulb. Six coronal slices were positioned for matching with NIRF images.

Computer-aided planimetric assessment of the lesion and hemispheric volumes were performed using image analysis software ImageJ (NIH, Bethesda, MD, USA). After adjustment of contrast, the contours of the hemispheres were traced manually on each slice. The position of the midline was determined using neuroanatomic landmarks as described previously [22]. Lesion volumes were determined by computer-aided manual tracing of the hyper-intense lesions and corrected for the space-occupying effect of brain edema as described previously using the following equation [22].

%HLV^e^ = (HVc−HVi+LV)/HVc*100; Where %HLV^e^ indicates edema–corrected lesion volume (in percent of the hemispheric volume); HVc and HV_i_ indicate contralateral and ipsilateral hemispheric volume; and LV indicates uncorrected lesion volume.

### Histological Examination

After ex vivo NIRF imaging, brain slices were incubated in a 2% TTC solution (Sigma Aldrich) at 37°C for 30 minutes. Hematoxylin and eosin staining using 6 µm thickness brain sections was performed to distinguish lesion border. The area of infarction was assessed by drawing an outline of the area of TTC pallor on scanned images of sections by an assessor blinded, and the volume of infarction was calculated without edema correction, to enable comparison with the ex vivo NIRF volumes.

### Immunohistochemistry

At each time point after injection of A15 probe, animals were anesthetized and transcardially perfused with heparinized saline followed by 4% of paraformaldehyde. After postfixation and dehydration, coronal sections (20 µm) of mouse brains were cut on a freezing microtome. A rabbit polyclonal antibody against fibrinogen/fibrin was used to detect the deposition of fibrin in brain (at dilution of 1∶500, Abcam, ab34629, Hong Kong). Sections were incubated with the primary antibody against fibrinogen/fibrin for 1 d at 4°C, followed by incubation with the secondary antibody (KPL, Kirkegaard & Perry Laboratories, Inc., NO. 02-15-06, MD, US) conjugated to FITC.

### Fluorescence Microscopy

A BX51 microscope with blue and red excitation filter units (Olympus, Japan) and CRi Nuance spectral imaging and quantifying system (Cambridge Research and Instrumentation Inc., Woburn, MA, USA) were used to examine the fluorescence signals in sections. All cubed image files were collected from the tissue slides at 10 nm wavelength intervals from 420 nm to 720 nm or from 670 nm to 720 nm with auto exposure times at 400× magnifications. Both mixed and separated FITC and Cy5.5 images were established after determining the FITC and Cy5.5 spectral library and unmixing the cubes. Quantitative colocalization analysis was performed using Image J (NIH, Bethesda, MD, USA).

### Statistical Analysis

Data are presented as mean ± SD. Comparisons were made using analysis of variance on ranks followed by 1-way ANOVA test, followed by Bonferroni post-test. Targeted signal values of in vivo NIRF images were plotted against ex vivo NIRF images or %HLV^e^, followed by a linear regression analysis to calculate R^2^ and to determine the regression equation. A value of P<0.05 was considered statistically significant.

## References

[1] Roger VL, Go AS, Lloyd-Jones DM, Adams RJ, Berry JD (2011). Heart disease and stroke statistics–2011 update: a report from the American Heart Association.. Circulation.

[2] Seshadri S, Beiser A, Kelly-Hayes M, Kase CS, Au R (2006). The lifetime risk of stroke: estimates from the Framingham Study.. Stroke.

[3] Durukan A, Tatlisumak T (2007). Acute ischemic stroke: overview of major experimental rodent models, pathophysiology, and therapy of focal cerebral ischemia.. Pharmacol Biochem Behav.

[4] Zhang RL, Chopp M, Zhang ZG, Jiang Q, Ewing JR (1997). A rat model of focal embolic cerebral ischemia.. Brain Res.

[5] Albers GW (1995). Antithrombotic agents in cerebral ischemia.. Am J Cardiol.

[6] Klohs J, Grafe M, Graf K, Steinbrink J, Dietrich T (2008). In vivo imaging of the inflammatory receptor CD40 after cerebral ischemia using a fluorescent antibody.. Stroke.

[7] Klohs J, Baeva N, Steinbrink J, Bourayou R, Boettcher C (2009). In vivo near-infrared fluorescence imaging of matrix metalloproteinase activity after cerebral ischemia.. J Cereb Blood Flow Metab.

[8] Hilderbrand SA, Weissleder R (2010). Near-infrared fluorescence: application to in vivo molecular imaging.. Curr Opin Chem Biol.

[9] van Dam GM, Themelis G, Crane LM, Harlaar NJ, Pleijhuis RG (2011). Intraoperative tumor-specific fluorescence imaging in ovarian cancer by folate receptor-alpha targeting: first in-human results.. Nat Med.

[10] Kasahara K, Souri M, Kaneda M, Miki T, Yamamoto N (2010). Impaired clot retraction in factor XIII A subunit-deficient mice.. Blood.

[11] Jaffer FA, Tung CH, Wykrzykowska JJ, Ho NH, Houng AK (2004). Molecular imaging of factor XIIIa activity in thrombosis using a novel, near-infrared fluorescent contrast agent that covalently links to thrombi.. Circulation.

[12] Tung CH, Ho NH, Zeng Q, Tang Y, Jaffer FA (2003). Novel factor XIII probes for blood coagulation imaging.. Chembiochem.

[13] Kim DE, Schellingerhout D, Jaffer FA, Weissleder R, Tung CH (2005). Near-infrared fluorescent imaging of cerebral thrombi and blood-brain barrier disruption in a mouse model of cerebral venous sinus thrombosis.. J Cereb Blood Flow Metab.

[14] Siebler M, Nachtmann A, Sitzer M, Steinmetz H (1994). Anticoagulation monitoring and cerebral microemboli detection.. Lancet.

[15] Heye N, Cervos-Navarro J (1996). Microthromboemboli in acute infarcts: analysis of 40 autopsy cases.. Stroke.

[16] Zhang ZG, Chopp M, Goussev A, Lu D, Morris D (1999). Cerebral microvascular obstruction by fibrin is associated with upregulation of PAI-1 acutely after onset of focal embolic ischemia in rats.. J Neurosci.

[17] Wang L, Kittaka M, Sun N, Schreiber SS, Zlokovic BV (1997). Chronic nicotine treatment enhances focal ischemic brain injury and depletes free pool of brain microvascular tissue plasminogen activator in rats.. J Cereb Blood Flow Metab.

[18] Tabrizi P, Wang L, Seeds N, McComb JG, Yamada S (1999). Tissue plasminogen activator (tPA) deficiency exacerbates cerebrovascular fibrin deposition and brain injury in a murine stroke model: studies in tPA-deficient mice and wild-type mice on a matched genetic background.. Arterioscler Thromb Vasc Biol.

[19] Mosesson MW, Siebenlist KR, Meh DA (2001). The structure and biological features of fibrinogen and fibrin.. Ann N Y Acad Sci.

[20] Klohs J, Steinbrink J, Nierhaus T, Bourayou R, Lindauer U (2006). Noninvasive near-infrared imaging of fluorochromes within the brain of live mice: an in vivo phantom study.. Mol Imaging.

[21] Okada Y, Copeland BR, Fitridge R, Koziol JA, del Zoppo GJ (1994). Fibrin contributes to microvascular obstructions and parenchymal changes during early focal cerebral ischemia and reperfusion.. Stroke.

[22] Gerriets T, Stolz E, Walberer M, Muller C, Kluge A (2004). Noninvasive quantification of brain edema and the space-occupying effect in rat stroke models using magnetic resonance imaging.. Stroke.

[23] Hara T, Mies G, Hossmann KA (2000). Effect of thrombolysis on the dynamics of infarct evolution after clot embolism of middle cerebral artery in mice.. J Cereb Blood Flow Metab.

[24] Ariens RA, Lai TS, Weisel JW, Greenberg CS, Grant PJ (2002). Role of factor XIII in fibrin clot formation and effects of genetic polymorphisms.. Blood.

[25] Muszbek L, Bagoly Z, Bereczky Z, Katona E (2008). The involvement of blood coagulation factor XIII in fibrinolysis and thrombosis.. Cardiovasc Hematol Agents Med Chem.

[26] Muszbek L, Bereczky Z, Bagoly Z, Komaromi I, Katona E (2011). Factor XIII: a coagulation factor with multiple plasmatic and cellular functions.. Physiol Rev.

[27] Miserus RJ, Herias MV, Prinzen L, Lobbes MB, Van Suylen RJ (2009). Molecular MRI of early thrombus formation using a bimodal alpha2-antiplasmin-based contrast agent.. JACC Cardiovasc Imaging.

[28] Uppal R, Ay I, Dai G, Kim YR, Sorensen AG (2010). Molecular MRI of intracranial thrombus in a rat ischemic stroke model.. Stroke.

[29] Adams RA, Passino M, Sachs BD, Nuriel T, Akassoglou K (2004). Fibrin mechanisms and functions in nervous system pathology.. Mol Interv.

[30] Ryu JK, Davalos D, Akassoglou K (2009). Fibrinogen signal transduction in the nervous system.. J Thromb Haemost.

[31] Kilic E, Hermann DM, Hossmann KA (1998). A reproducible model of thromboembolic stroke in mice.. Neuroreport.

